# Experimental Study on the Curing Mechanism of Red Mud-Based Stabilized Soil Co-Modified by Nano-SiO_2_ and Gypsum

**DOI:** 10.3390/ma16176016

**Published:** 2023-09-01

**Authors:** Shengjin Chen, Xiaoduo Ou, Jie Jiang, Zhijie Tan

**Affiliations:** 1 Guangxi Hualan Geotechnical Engineering Co., Ltd., Nanning 530016, China; chenshengjin@gxhlyt.cn (S.C.); wgytzj@163.com (Z.T.); 2 School of Civil Engineering and Architecture, Guangxi University, Nanning 530004, China; jie_jiang001@126.com

**Keywords:** red mud, nano-SiO_2_, synergistic modification, stabilized soil, curing mechanism

## Abstract

In order to effectively utilize red mud and reduce its occupation of land resources, as well as its impact on the environment, experiments were conducted to develop stabilized soil materials using nano-SiO_2_ synergistically modified red mud and to investigate the curing mechanism of stabilized soil. The unconfined compressive strength, microscopic morphology, and curing mechanism of the red mud-based stabilized soil materials with different amounts of modified materials were investigated. The test results show that after 7 days of curing, the unconfined compressive strength of red mud-based stabilized soil meets the compressive strength requirement of road base material when nano-SiO_2_, gypsum, and cement are synergistically modified. In such cases, the soil structure has the lowest fracture rate and the best structural compactness when the amount of nano-SiO_2_ is 1%. It is found that the needle-like and columnar calcium alumina in the modified red mud-based stabilized soil increases, and the binding energy of hydration product ions in the modified material is improved. The chemical curing mechanism of modified red mud-based stabilized soil includes hydration reaction, pozzolanic reaction, promotion effect of nano-SiO_2_, and enhancement effect of gypsum. On this base, a model of the early start hydration process of red mud-based stabilized soil promoted by nano-SiO_2_ is established.

## 1. Introduction

Red mud, which is of high iron oxide content and red color, is a solid waste generated during the process of alkali alumina production. According to relevant statistics, for every 1 ton of alumina produced, 1 to 2 tons of red mud is generated [1,2]. Currently, red mud is disposed of by open damming and stockpiling. The utilization of red mud is strictly controlled [3,4], making the utilization rate of red mud extremely low (less than 10% utilized so far) [5,6], and red mud has become one of the bulk industrial solid wastes. Therefore, it is of practical significance to change the treatment of red mud from simple stockpiling to resource-based integrated utilization.

Red mud can be used to make ceramics and bricks [7,8,9], is used as raw material for cement production [10], is applied to road base materials, etc. Considering that road base materials can consume large amounts of red mud [11,12,13], currently, the research on the application of red mud in engineering is intense and has shown great potential [14,15,16,17]. Mukiza et al. 6] investigated the possibility of using red mud as a road-base material, showing that the synergistic use of red mud and other wastes can improve the mechanical properties and durability performance of the material compared to red mud alone. Chandra et al. [18] developed geopolymer road base material using red mud, fly ash, and alkaline activator solution and found that the material had CBR values up to 12% and UCS up to 2700 kPa. The microstructure of the material showed that the formation of dense calcium aluminate hydrate (C-A-H) and calcium silicate hydrate (C-S-H) was responsible for the increase in strength. Ou et al. [13] used red mud and bauxite tailing mud to develop roadbed materials and found that the strength of the materials mainly originated from the hydration and pozzolanic reaction, while the gelling products generated from the hydration and pozzolanic reaction solidified Na+ and inhibited the release of OH^−^. Satayanarayana et al. [19] stabilized red mud using lime and found that red mud with 10% lime added had a CBR of 25% at 28 days of age, which can be used as a sub-base and base material in road construction. Zhang et al. [20] prepared road base material using electrolytic manganese slag-red mud-electrolytic slag as the main raw material, and the results showed that the road base material hydrated to produce C-A-S-H gel and calcium alumina, and the road base material had high unconfined compressive strength and good durability. Through the above literature, it can be seen that scholars have conducted in-depth research on the feasibility of the red mud road base material and also analyzed its causal mechanism in depth.

Nanomaterials are extremely small particles with a particle size of 1 to 100 nm, with high specific surface area and good volcanic ash activity [21]. Scholars have introduced nano-SiO_2_ materials into the field of civil engineering, and their main applications are the improvement of concrete materials and soils [22,23,24,25]. Ahmed et al. [26] incorporated nano-SiO_2_ in the synthesis of geopolymer binder with red mud as the main solid source and found that nano-SiO_2_ contributed to the formation of C-S-H in the geopolymer and increased the compressive strength. The performance advantages of nano-SiO_2_ are obvious, but how to utilize the advantages of nanomaterials and exploit them in the modification of red mud into roadbed materials is rarely studied. Therefore, in this paper, nano-SiO_2_ is synergistically involved in the modification of red mud-based stabilized soil, and the mechanical properties and microstructure of the stabilized soil are experimentally studied. Based on the obtained results, the curing mechanism of nano-SiO_2_ synergistically modified red mud-based stabilized soil is analyzed.

## 2. Materials and Methods

### 2.1. Raw Materials

#### 2.1.1. Red Mud

The red mud used in the test was taken from an aluminum company in Guangxi Province, which is Bayer red mud. The chemical composition of the red mud is shown in Table 1. It shows that the red mud contains 5.23% Na_2_O, which can provide hydroxide for the hydration process.

#### 2.1.2. Nano-SiO_2_

The nano-SiO_2_ used was produced by the mechanical crushing process. Nano-SiO_2_ is in powder form with a large specific surface area and high surface activity. Its technical parameters are listed in Table 2.

#### 2.1.3. Cement

The cement used in the test is 42.5 standard ordinary silicate cement. It has a specific surface area of 340 m^2^/kg and a density of 3.10 g/cm^3^. The chemical composition is shown in Table 3.

It shows that the main mineral composition of the cement comprises tricalcium silicate 3CaO-SiO_2_ (C3S), accounting for 50–60%, dicalcium silicate 2CaO-SiO_2_ (C2S), accounting for 20–25%, called Belite or B ore, tricalcium aluminate 3CaO-AlO_23_ (C3A), accounting for 5–10%, and tetra calcium iron aluminate, 4CaO-AlO_23_-FeO_23_ (C4AF), accounting for 10–15%.

#### 2.1.4. Gypsum

According to the Chinese national standard of Calcined gypsum (GB/T 9776-2022) [27], the selected gypsum is a kind of construction gypsum produced by Jinan Desheng Chemical Technology Co., Ltd., Jinan, China. It is a kind of white powder with a density of 2.32 g/cm^3^. Its chemical formula is CaSO_4_-0.5H_2_O. Samples need to be made at a controlled time because of the fast setting time of the gypsum.

### 2.2. Specimen Preparation

When stabilized soil is applied to the subgrade material, the strength requirements need to be met first. Referring to Table 4 in Technical Guidelines for Construction of Highway Roadbases (JTG/T F20-2015) [28], the 7 d unconfined compressive strength of red mud-based stabilized soil should be greater than or equal to 2 MPa to serve as the base material used for medium and light traffic secondary and secondary roads.

Combining the studies of Gayathiri et al. [29] and Xue et al. [4], the designed mass ratios added in the red mud-based stabilized soil of nano-SiO_2_ are 0.5%, 1%, 1.5%, 2%, 2.5%, and 3%. The addition of gypsum in the modified material can continuously provide Ca^2+^ for the red mud liquid phase system, promote the generation of Aft and C-S-H, C-A-H, improve the strength of the material, and, at the same time, reduce the OH^−^ content of the red mud and lower the pH value (when gypsum mixing ≥ 5%). Considering the need for strength and alkali control, the gypsum was mixed with the red mud-based stabilized soil according to the mass ratio of 6%. Cement was at 1%, 3%, 5%, 7%, and 9%; considering the economic benefits and reduction in carbon emissions, the optimal dosage of cement was the minimum dosage that met the strength requirements. 

The specimen preparation is as follows:

① Drying and grinding of red mud specimens into powder form;
② Add cement and powdered gypsum to the red mud specimen with the designed dosage and use a small mixer to mix well;
③ Add deionized water to the specimen with the maximum dry density and optimum water content determined by the compaction test and mix well;
④ The test material will be made into 5 mm × 5 mm cylindrical compressive specimens, and the specimen production time should be controlled within 20 min after adding gypsum;
⑤ The finished specimens were placed in an environment with a temperature of 20 °C and a relative humidity of 95% for curing, and on the last day of the curing time, the specimens were immersed for 24 h, after which the specimens were taken out and air-dried, and the unconfined compressive test was performed;
⑥ After the specimens are made, they are left to standing curing (in an environment with a temperature of 20 °C and a relative humidity of 95%) for different periods (i.e., 1, 7, 14, 28, 60, and 120 days) before the mechanical and microstructure experiments. On the last day of the curing time, the specimen will be immersed in water for 24 h and then removed and air-dried.

According to the above preparation steps, stabilized soil specimens with different contents of red mud, cement, and nano-SiO_2_ were prepared. The synergistic combination scheme of the added stabilizers is shown in Table 4, where NS1CS6PC3 indicates that the doping mass ratio of nano-SiO_2_ is 1%, the doping mass ratio of gypsum is 6%, and the doping mass ratio of cement is 3%.

### 2.3. Testing Methods

#### 2.3.1. Unconfined Compression Test

**Figure 1:**
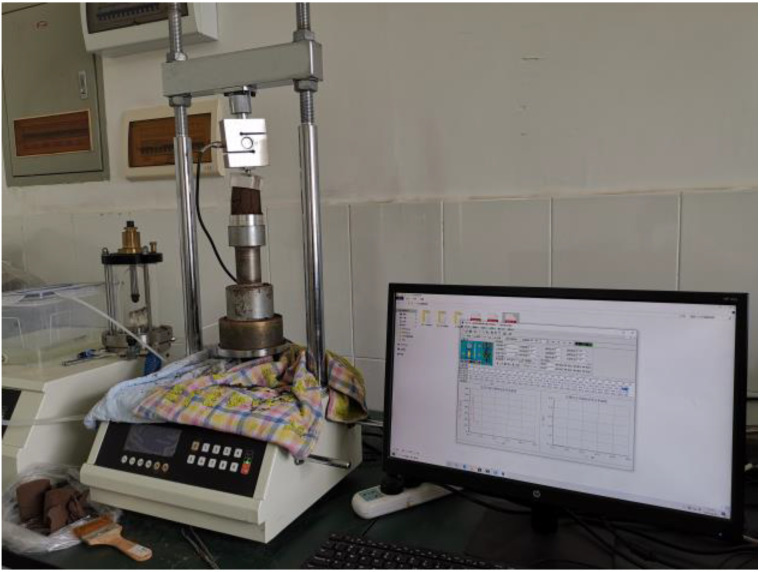
The TSZ series fully automatic triaxial instrument.

The compression was conducted under the condition that the unconfined compression test was carried out using the TSZ series automatic triaxial instrument (Figure 1) produced by Nanjing Soil Instrument Factory Co. (Nanjing, China). This instrument can directly obtain the experimental maximum compression strength and the relationship curve between the main stress difference and axial strain. The unconfined compressive strength test is based on the Chinese industry standard Test Methods of Materials Stabilized with Inorganic Binders for Highway Engineering (JTG-E51-2009) [30]. During the test, the loading rate was kept at 1 mm/min.

#### 2.3.2. Scanning Electron Microscope Test

A scanning electron microscope (SEM) of type S-3400 produced by Hitachi, Tokyo, Japan, was used for microstructure observation, which has a magnification of 20~30,000 times. Before observation, the dried specimen is cut into observation cubes with dimensions of 4 mm (length) × 4 mm (width) × 2 mm (thickness) using a geotechnical knife. The observation specimens are connected to the metal panels for observation through conductive strips, and then, the metal panels are transferred to the Ion Sputter Giko IB-3 gold spraying instrument manufactured by Shanghai Hezao Electronics Company in Shanghai, China, for gold spraying treatment with a thickness of 20–30 μm. The microstructure of the samples is obtained using SEM in backscattering mode at an accelerating voltage of 5 kV.

#### 2.3.3. Energy Spectrum Analysis Test

Energy spectrometry (EDX) is the analysis of all elemental species and contents between Be-U within the micro-zone of the material using an energy dispersive spectrometer, which is used in conjunction with a scanning electron microscope.

#### 2.3.4. X-ray Diffraction Test

X-ray diffraction (XRD) tests are used to measure the mineral composition of test materials. Before testing, the red mud-based stabilized soil material was oven-dried, after which the samples were ground into powder form and set aside. The mineral composition was qualitatively analyzed using an X’Pert PRO MRD/XL high-resolution diffractometer with CuKa radiation, manufactured by PANalytical, Almelo, the Netherlands. During these experiments, the ray wavelength λκα was 1.54060 Å; the tube pressure was 40 KV; the tube electric current was 40 mA; the scanning range was 5° to 75°; the step size was 0.02°, and the scanning speed was 5°/min.

#### 2.3.5. X-ray Photoelectron Spectroscopy Test

X-ray photoelectron spectroscopy (XPS) is an advanced analytical technique in the microscopic analysis of electronic materials and components. Before the test, the red mud-based stabilized soil material was oven-dried, after which the sample was ground into powder form and set aside. A PHI-5300ESCA X-ray photoelectron spectrometer, manufactured by Palo Alto, CA, USA, was used for XPS analysis of the red mud-based stabilized soil specimens with an Mg/Al anode target with 400 W power and the analyzer charge set to 17.5 eV, which was detected by a position-sensitive detector.

## 3. Results and Analysis

### 3.1. The Unconfined Compressive Strength

#### 3.1.1. Effect of Cement Content

**Figure 2:**
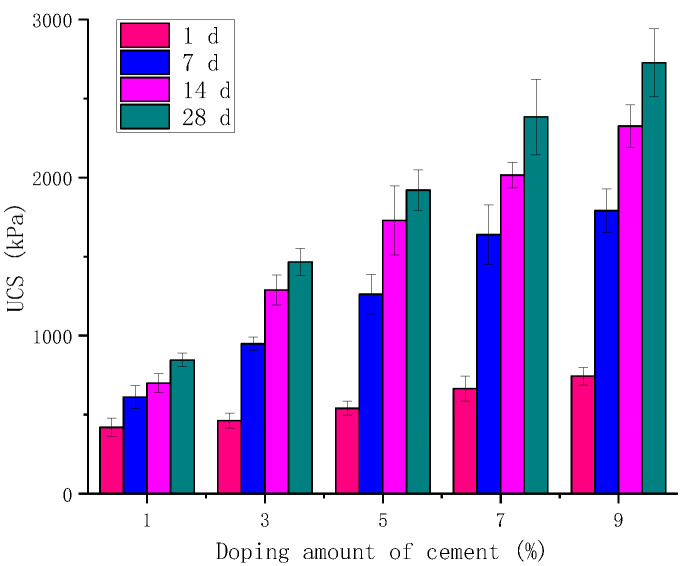
Effect of cement on UCS.

The unconfined compression tests were conducted on the cylindrical specimens of red mud-based stabilized soil mixed with cement alone at 1%, 3%, 5%, 7%, and 9%, respectively. The test results are shown in Figure 2.

Cement-modified red mud-based stabilized soil has higher compressive strength with increasing cement admixture; the unconfined compressive strength of the cement-modified red mud-based stabilized soil was 491 kPa, 928 kPa, 1262 kPa, 1639 kPa, and 1797 kPa for the specimens with 1%, 3%, 5%, 7%, and 9% cement dosing, respectively.

The unconfined compressive strength of cement-modified red clay-based stabilized soil increased with the increase in curing time, but the increase in unconfined compressive strength from 1 d to 7 d was greater than the increase in curing time from 7 d to 14 d and that from 14 d to 28 d. The increase in unconfined compressive strength of cement-modified red clay-based stabilized soil was calculated as the increase in curing time from 1 d to 7 d and from 14 d to 28 d only. It is assumed that the presence of soluble alkali in the red mud can promote the hydration reaction, and the strength increase in the stabilized soil by the modification of cement is also mainly concentrated in the pre-curing period.

#### 3.1.2. Effect of Synergistic Modification of Nano-SiO_2_, Gypsum, and Cement

**Figure 3:**
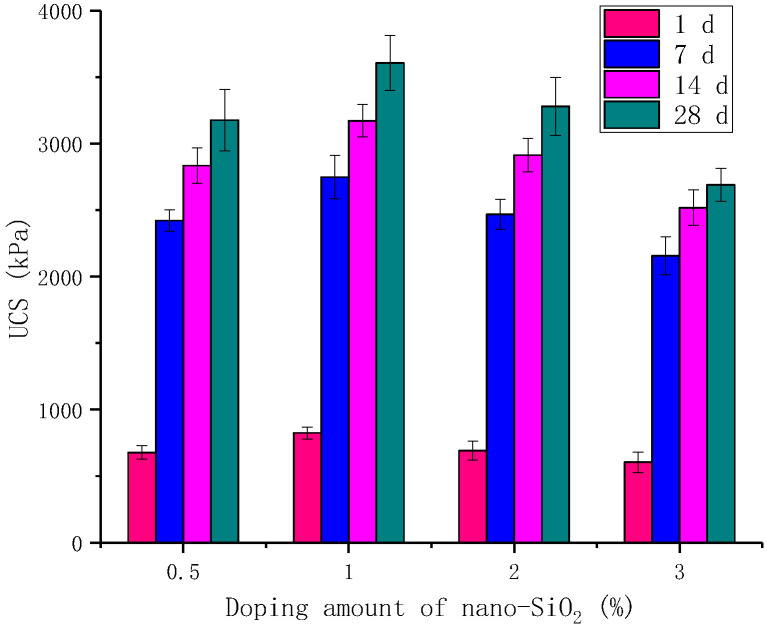
Effect of nano-SiO_2_ content on UCS of gypsum and cement synergistic combination.

To verify the optimum dosing of nano-SiO_2_ in the modified material, the dosing of gypsum was chosen to be 6% in this section; the dosing of cement was chosen to be 3% considering the economy, and the dosing of nano-SiO_2_ was chosen to be 0.5%, 1%, 2%, and 3% for the tests. Figure 3 shows the relationship between the unconfined compressive strength of the modified material and the variation of nano-SiO_2_ dosing. It can be found that the unconfined compressive strength did not increase with the increase in nano-SiO_2_ dosing, and the unconfined compressive strength was maximum at 1% dosing. For example, the unconfined compressive strength at 7 d curing time is 2421, 2748, 2467, and 2156 kPa for 0.5%, 1%, 2%, and 3% of nano-SiO_2_, respectively. They all satisfy the condition of 7 d unconfined compressive strength of red mud-based stabilized soil as proposed in Section 2.2.

Figure 3 shows that the unconfined compressive strength of the modified red mud-based stabilized soil increased with the increase in the maintenance time, and the growth rate of the unconfined compressive strength was the largest from 1 to 7 d in the early stage. In the case of a 1% dosing of nano-SiO_2_, the growth value of the first 7 d compressive strength accounted for 69.2% of the growth value of the 28 d compressive strength. The highest unconfined compressive strength of the modified combination can be obtained at 1% of nano-SiO_2_. Hou et al. [31] concluded that when the nano-SiO_2_ doping is too much, the nano-SiO_2_ tends to form agglomerates and adsorb water, encroaching on the water for hydration reaction, affecting the degree of hydration and causing the strength of the red mud-based stabilized soil to decrease, so the NS2CS6PC3 and NS3CS6PC3 combinations have lower unconfined compressive strengths than the NS1CS6PC3 combination.

**Figure 4:**
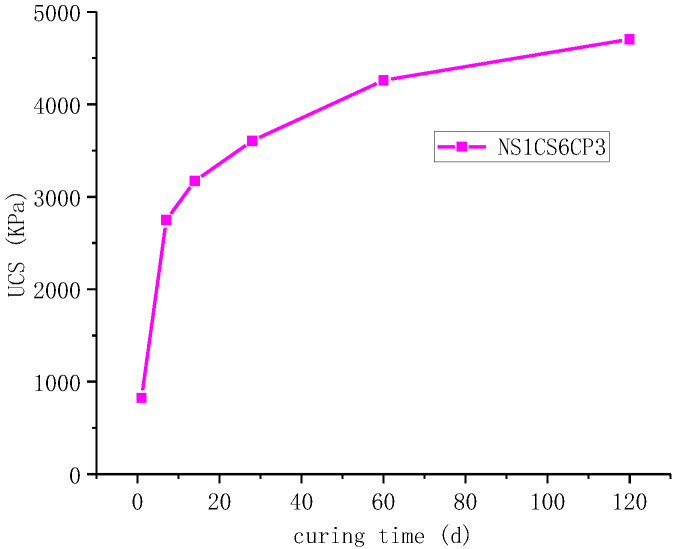
Curve of UCS of NS1CS6PC3 combination with curing time.

Figure 4 shows that the age of 7 d was the inflection point of unconfined compressive strength growth rate, and the growth rate of unconfined compressive strength gradually decreased after the age of 7 d, and the average growth rate of unconfined compressive strength was 7.4 kPa/d during 60 d~120 d.

### 3.2. Micro-Morphology and Curing Characteristics of Red Mud-Based Stabilized Soil

#### 3.2.1. Micromorphology

**Figure 5:**
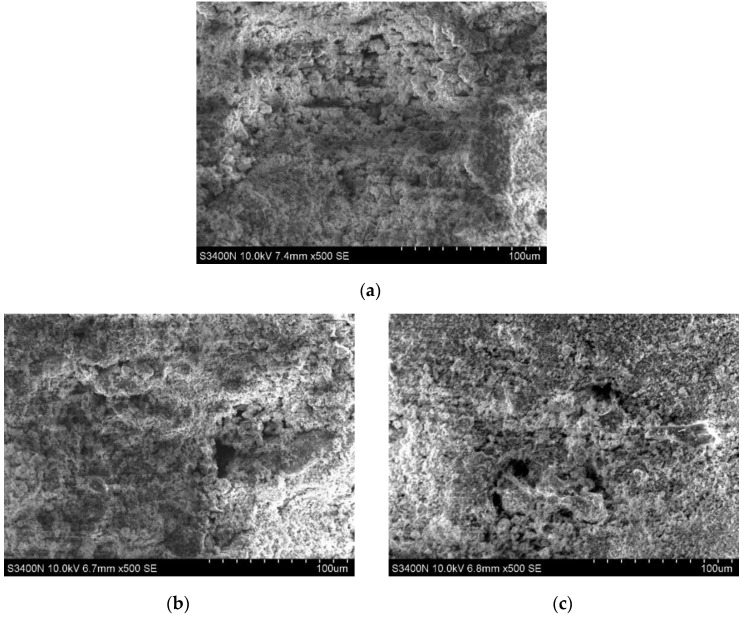
The 500-time SEM images of red mud stabilized soil modified by nano-SiO_2_, gypsum, and cement. (**a**) nano-SiO_2_ at 1%; gypsum at 6%, and cement at 3%. (**b**) nano-SiO_2_ at 2%; gypsum at 6%, and cement at 3%. (**c**) nano-SiO_2_ at 3%; gypsum at 6%, and cement at 3%.

The gypsum dose of 6%, the cement dose of 3%, and the nano-SiO_2_ dose of 1%, 2%, and 3%, respectively, were selected for electron microscope scanning at 28 d. The observation magnification of the electron microscope used in the test was 500 times. The SEM images after the test are shown in Figure 5. It shows that the structural compactness of the soil did not increase with the increase in nano-SiO_2_ under the condition of a certain amount of gypsum and cement admixture. On the contrary, it showed a decreasing trend, which indicates that in the synergistic modification combination of nano-SiO_2_, gypsum, and cement, there exists a limit for the amount of nano-SiO_2_. When the admixture of nano-SiO_2_ is 1%, the surface of the soil is relatively flat; the pore development is less; the fracture rate is low, and the soil structural compactness is relatively good.

#### 3.2.2. Curing Characteristics

(1) SEM-EDX

**Figure 6:**
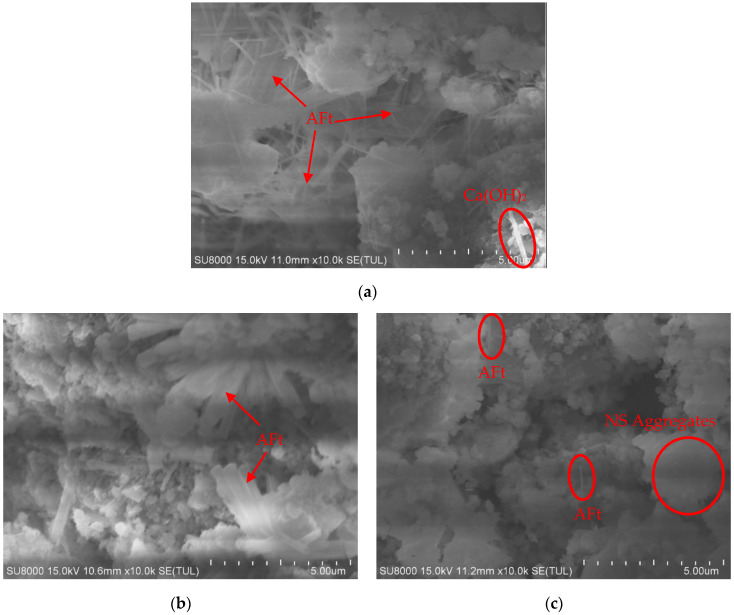
The 10,000-time SEM images of red mud stabilized soil modified by nano-SiO_2_, gypsum, and cement. (**a**) nano-SiO_2_ at 1%; gypsum at 6%, and cement at 3%. (**b**) nano-SiO_2_ at 2%; gypsum at 6%, and cement at 3%. (**c**) nano-SiO_2_ at 3%; gypsum at 6%, and cement at 3%.

The gypsum dose of 6%, the cement dose of 3%, and the nano-SiO_2_ dose of 1%, 2%, and 3%, respectively, were selected for electron microscope scanning at 28 d. The observation magnification of the electron microscope used in the test was 10,000 times. The SEM images after the test are shown in Figure 6.

The content and morphology of some gelling products in the 10,000× electron microscope scan images differ. Figure 6a shows that the gelling products are mainly needle-like and columnar calcium alumina (AFt), and the AFt is longitudinally and horizontally distributed between particles or agglomerates, interconnected and interwoven, forming a huge spatial mesh structure to support the material skeleton system, while Ca(OH)_2_ is occasionally seen to be distributed. Figure 6b shows that the gelling products are mainly columnar AFt, which is in clusters. Figure 6c shows that the gelling products are not obvious, the distribution of needle-like AFt is occasionally seen, and the red mud agglomerates are also found. It is assumed that the excessive nano-SiO_2_ fills and blocks the pores and channels of the stabilized soil, which reduces the migration of ions, such as OH^−^, Ca^2+^, and Al^3+^, and hinders the generation of AFt and other products.

The synergistic participation of gypsum promotes more AFt generation, which is more obvious at nano-SiO_2_ doping of 1% and 2%. AFt is generated by the reaction between gypsum and tricalcium aluminate in cement clinker with the reaction equation 3C3A + 3(CaSO_4_-2H_2_O) + 26H_2_O → 3CaO-AlO_23_-3CaSO_4_-32H_2_O. Usually, the percentage of gypsum in silicate cement is lower than 3%, while 6% of gypsum is added to this group of modified materials, which makes the red mud-based stabilized soil materials have the conditions to generate more AFt.

**Figure 7:**
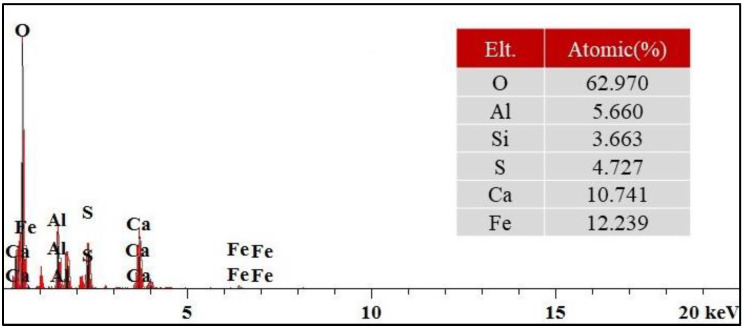
EDX energy spectrum of NS1CS6PC3 modified composite aggregate.

Figure 7 shows the EDX energy spectrum of the NS1CS6PC3 modified combined agglomerates. Compared with the EDX energy spectrum of pure red mud, the presence of S elements within the stabilized soil material indicates that gypsum intervenes in the reaction, and the material has a higher content of O and Ca elements; presumably, more AFt is generated within the modified material.

(2) XRD

**Figure 8:**
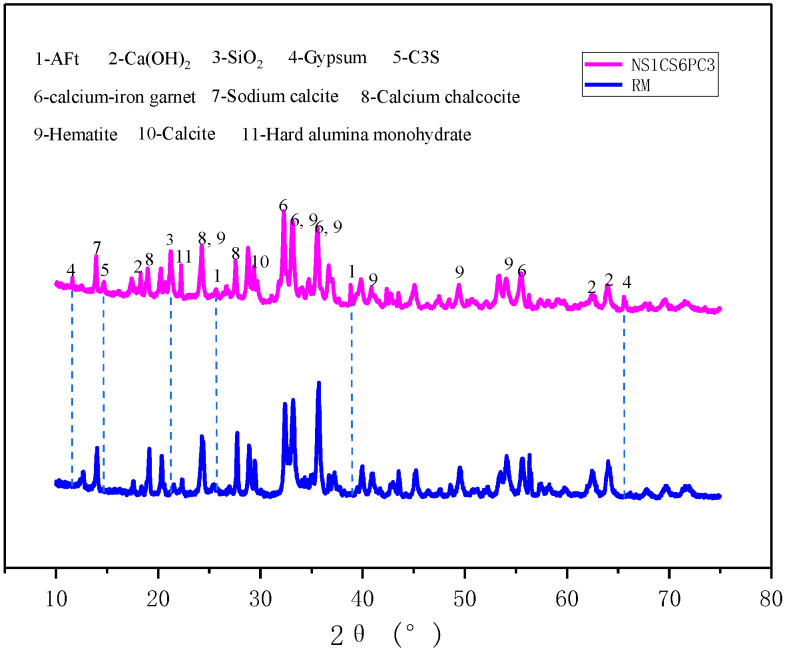
XRD patterns of NS1CS6PC3-modified red mud stabilized soil and red mud.

Figure 8 shows that the XRD pattern of modified red mud-based stabilized soil is similar to that of red mud in terms of peak area, peak size, and peak trend, mainly because the amount of modified materials in red mud-based stabilized soil is very small, and the new material generation should be relatively limited. However, due to the addition of the modified materials, new products were generated, so their XRD patterns had peaks that were not present in the red mud XRD pattern.

The XRD pattern of the modified red mud-based stabilized soil contains physical phases of hematite, calcium-iron garnet, sodium calcite, calcium chalcocite, and calcite in red mud and also contains physical phases of AFt, silica, gypsum, and tricalcium silicate, which are not present in red mud, where AFt is a hydration product, and tricalcium, gypsum, and silica are incompletely reacted modified materials. The peak of Ca(OH)_2_ in the modified red mud spectrum overlaps with the red mud, and the analysis of this peak may be the physical phase of portlandite in the red mud, and the main peak does not see the hydrated Ca(OH)_2_ physical phase. According to the analysis, after the hydration reaction between the cement with little admixture and the water contact in the modified material, the hydrated Ca(OH)_2_ is generated, but the alumina and silicon oxide, which exist in large quantities in the red mud, react with the hydrated Ca(OH)_2_ of cement under the alkaline environment conditions in a pozzolanic reaction, and the Ca(OH)_2_ generated by its hydration is also small due to the little admixture of the cement, then the alumina and silicon oxide, which exist in large quantities in the red mud, gradually consume the hydrated Ca(OH)_2_. The cement hydration products, C-S-H gels and C-A-H gels, have no peaks in the XRD patterns because of their non-crystalline structure.

(3) XPS

The binding energy refers to the mutual attraction between the components within an object that binds them together, and if one wants to separate these components, a certain amount of energy is required to overcome the attraction between them, which is the binding energy of the object, and the amount of work required indicates the tightness of the combination of these components. The greater the binding energy, the greater the attraction or cohesion between the components of the material.

Based on the XPS energy spectra of the red mud-based stabilized soil materials with different modification combinations and different hydration ages, the binding energies of the main elements, such as Ca, Si, Al, Na, O, and S, during hydration can be derived, as shown in Table 5.

As shown in Table 4, in the same set of modified materials, the binding energy of the main elements, such as Ca, Si, Al, Na, O, and S, in the red mud-based stabilized soil materials are increasing with the increase in the maintenance age, which indicates that the free Ca, Si, Al, Na, O, and S elements in the liquid phase of the red mud-based stabilized soil materials continuously participate in the hydration reaction with the increase in the maintenance age and generate Ca(OH)_2_, C-S-H gels, Aft, and other hydration products, while new covalent and ionic bonds are formed in this process, which makes the binding energy of each element enhanced [32].

However, the increase or decrease in the binding energy of each major element with different doping amounts of nano-SiO_2_ did not exactly show a positive or negative correlation with the amount of nano-SiO_2_. It is speculated that this phenomenon is related to the elemental properties and the calcium–silicon ratio.

## 4. Curing Mechanics of Red Mud-Based Stabilized Soil

We analyze the properties and change mechanisms of red mud-based stabilized soils by SEM-EDX, MIP, XRD, and XPS and conclude that the curing mechanisms of red mud-based stabilized soils include hydration reactions, pozzolanic reactions (secondary hydration reactions), the promotion effect of nano-SiO_2_, and the enhancement effect of gypsum, which are chemical reactions. In the case of stabilized soil bulk materials, the premise for these reactions is that the materials first undergo a physical process—mechanical compaction—and when mechanically compacted, the bulk materials are tightly joined together so that they have the contact conditions for chemical reactions to occur between them. The curing mechanisms are independent of each other but are interconnected, and the common interconnection forms a synergistic curing mechanism that promotes the formation of the strength of the red mud-based stabilized soil.

### 4.1. Mechanical Compaction

Through mechanical compaction, the friction and embedded force between the particles of the red mud-based stabilized soil material formed, and the friction and embedded force between the particles contributed to the macroscopic mechanical strength, which is the mechanism of mechanical compaction to obtain the strength of the material. Once the particles of the red mud-based stabilized soil material are in close contact with each other through mechanical compaction and have gained initial strength, the chemical reaction between the mixes continues to occur as the maintenance time increases, generating cementing substances that result in tighter particle bonding and higher structural strength.

### 4.2. Hydration Reaction and Pozzolanic Reaction

The modified material in the red mud-based stabilized soil contains cement clinker, which is highly reactive. When it meets water, each component of the cement dissolves rapidly and undergoes a hydration reaction, which is the main reason for the strength of red mud-based stabilized soil. The hydration reaction of cement is a series of reactions of tricalcium silicate (C3S), dicalcium silicate (C2S), tricalcium aluminate (C3A), and tetra calcium iron aluminate (C4AF) in cement with the participation of water, and the process of hardening strength of the material is improved by the generation of cementitious products.

Pozzolanic reaction, also known as secondary hydration reaction, refers to the process that active components such as SiO_2_ and Al_2_O_3_ in minerals react with Ca(OH)_2_ in an alkaline environment to produce gelling products such as C-S-H, C-A-H, AFt, etc. The proportion of SiO_2_ and Al_2_O_3_ in red mud is 12.35% and 18.89%, respectively, and the active component accounts for a relatively large proportion; nano SiO_2_ soluble in alkali, in the alkaline environment belongs to the active material, can provide Si^4+^; gypsum can provide additional Ca^2+^; red mud soluble alkali such as NaOH, KOH can provide OH^−^ in addition to cement hydration and then supplement OH^−^. Therefore, the modified red mud-based stabilized soil itself already has good conditions for the pozzolanic reaction. The pozzolanic reaction is an important reason for the late strength growth of red mud-based stabilized soil.

### 4.3. Facilitating Effect of Nano-SiO_2_

The synergistic participation of nano-SiO_2_ improves the strength of the red mud-based stabilized soil. The contribution of nano-SiO_2_ is reflected in the promotion of the early hydration process and hydration degree through the “nucleation effect” on the one hand, the provision of more active silicon sources on the other hand, and the generation of more cementation products under alkaline environment and the reduction in material porosity and improvement of material compactness through the filling effect.

#### 4.3.1. Promotion of Early Hydration Reaction

Nano-SiO_2_ with a high specific surface area and good volcanic ash activity improves concrete properties. The high volcanic ash activity accelerates the hydration of the nano-SiO_2_-cement system to form C-S-H gels and also fills the pores of the concrete, which improves the early strength of the concrete [28,33]. According to Land et al. [34], the high specific surface area of nano-silica and its high activity in the alkaline environment make it have a “crystalline nucleation effect”, and the nano-silica uniformly distributed in the red mud-based stabilized soil acts as a “nucleation crystal species”, providing nucleation sites, allowing for more hydration ions to gather and react in the periphery, accelerating the early hydration process of the red mud-based stabilized soil, promoting the early production of more hydration products, and improving the strength of the red mud-based stabilized soil. Lin et al. [35] added nano-SiO_2_ to limestone calcined clay cements, and the results showed that nano-SiO_2_ significantly improved the strength of limestone calcined clay cements, accelerated their reaction rate, and greatly improved their early strength. The nano-SiO_2_ can refine its microscopic pore structure, improve the carbonation resistance, and enhance long-term performance.

**Figure 9:**
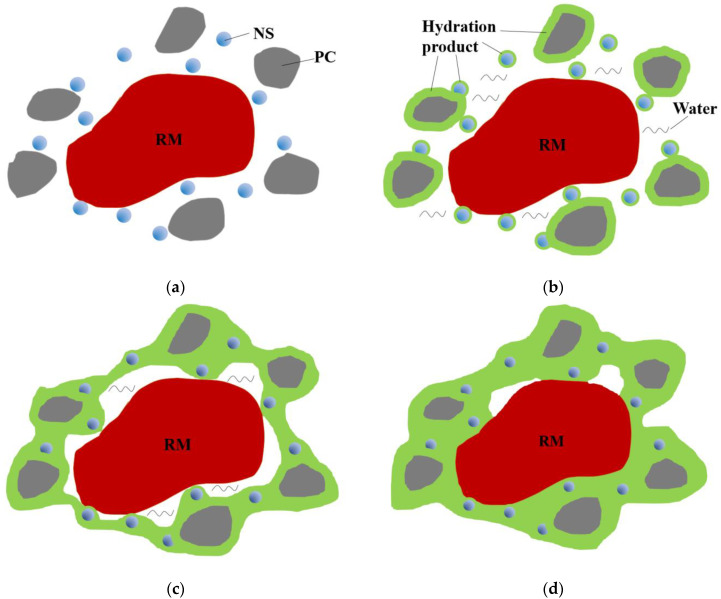
Model diagram of nano-SiO_2_ synergistic promoting hydration process. (**a**) uniform distribution of dry material after mixing. (**b**) Addition of water and generation of hydration products. (**c**) Continuous generation and interconnection of hydration products. (**d**) Hydrolysis products wrapped around red mud particles.

Combining the properties of nano-SiO_2_, the model of nano-SiO_2_ promoting the early hydration process of the red mud-based stabilized soil was established in this study with individual red mud particles, see Figure 9.

In the first step, as shown in Figure 9a, the ground red mud in powder form is mixed with the modified material in a dry state so that the material particles are uniformly distributed. The red mud particles are surrounded by the cement, while the smaller particle size nano-SiO_2_ is filled between the red mud and cement particles.

In the second step, as shown in Figure 9b, water is added to the mixed particles in the designed proportion, and the hydration reaction occurs rapidly after the water is added and the hydration products are formed around the cement particles, while the “nucleation effect” formed by the nano-SiO_2_ due to its high surface energy makes the hydration ions of cement also gather around it rapidly, which, in turn, makes the hydration products also be formed around it and gathered around it.

In the third step, as shown in Figure 9c, with the hydration reaction, the hydration products are continuously generated. The original state of large pores between cement particles due to the small amount of cement, and the hydration products are not easily connected in series; due to the dispersed presence of nano-SiO_2_ and the continuous gathering of hydration products around themselves, the cement particles can be continuously connected and bonded by nano-SiO_2_ and hydration products.

In the fourth step, as shown in Figure 9d, the hydration reaction continues, and the hydration products keep connecting to fill the particle voids, and finally, a network body is formed around the red mud particles, which wraps the red mud particles and also serves to bond the different red mud particles, thus improving the overall compactness and structural strength of the red mud-based stabilized soil. The variation of this step can be better explained by the staggered distribution of Aft and wrapping of red mud particles shown in Figure 6a.

The content of C3S is about 50–60% in cement, which is the main contributor to the early strength of cement; then, the promotion effect of nano-SiO_2_ is mainly concentrated in the early stage during the synergistic process of nano-SiO_2_, which is the mechanism of nano-SiO_2_ to promote the early hydration reaction.

#### 4.3.2. Provide Silicon Source

Nano-silica is stable but soluble and reactive under alkaline environmental conditions, so the synergistic participation of nano-SiO_2_ continuously replenishes the liquid-phase system with Si^4+^ ions, providing more silicon sources for the red mud-based stabilized soil. Si^4+^ provided by nano-SiO_2_ and Ca^2+^ together with OH^−^ ions (generated by cement hydration and by soluble alkali in red mud), in turn, undergo secondary hydration reactions to generate more C-S-H, C-A-H, AFt, and other gels, further enhancing strength. The red mud acts as a “hotbed”, and the alkali environment provided by it allows the relatively stable nano-SiO_2_ and gypsum to react continuously, but the reaction is more moderate and can last for a longer period.

### 4.4. Enhancement Effect of Gypsum

The synergistic involvement of CS in the modified material, likewise, significantly improved the mechanical strength of the red mud-based stabilized soil. The gypsum was blended at 6%, and in an alkaline environment, gypsum was gradually dissolved in the liquid phase, which could continuously provide Ca^2+^ ions to the liquid phase system. During the hydration of cement, tricalcium aluminate C3A reacts with Ca(OH)_2_ and gypsum to form trisulfate hydrated calcium sulfate (AFt), and simply in the cement system, if gypsum is insufficient and consumed before C3A is fully hydrated, the trisulfate hydrated calcium sulfate will again react with the unconsumed C3A to convert into monosulfate hydrated calcium sulfate (AFm). According to Hargis [36], the strength of AFt or its contribution to material strength is greater than that of AFm, so once AFt is converted to AFm, it will have a negative impact on material strength. Since the red mud-based stabilized soil modification material has an additional 6% gypsum co-added in addition to 3% cement, the amount of gypsum is sufficient for the hydration reaction, and it is presumed that all or the vast majority of the end product of C3A in cement is AFt, which enhances the strength of the modified stabilized soil. Figure 6a shows the characteristics.

### 4.5. Effect of Calcium to Silicon Ratio

In the alkaline environment of red mud, the modified material and the active ingredients in red mud are excited and chemically react to produce more cementation products and promote strength. Different materials have different calcium–silica ratios, and different calcium–silica ratios have different effects on material strength.

Calculation of calcium to silicon ratio based on Equation (1):

(1) $$\frac{C}{S}=\frac{CaO\;total\;content÷56}{SiO_{2}\;total\;content÷60}$$

where the total calcium oxide content = ∑ material ratio × calcium oxide content, and the total silicon oxide content = ∑ material ratio × silicon oxide content.

The calculated calcium–silica ratios of NS1CS6PC3, NS2CS6PC3, and NS3CS6PC3 were 2.83, 2.55, and 2.32, respectively, which were higher than 0.92 for red mud and lower than 3.23 for cement, and the addition of modified materials improved the calcium–silica ratio of red mud, and the calcium–silica ratio was close to that of cement. Section 3.1.2 revealed that the unconfined compressive strengths of NS1CS6PC3, NS2CS6PC3, and NS3CS6PC3 at the age of 7 d were 2748, 2467, and 2156 kPa, respectively, indicating that the unconfined compressive strength increased with the increase in calcium–silica ratio in the three groups of modified materials. It indicates that the increase in calcium–silica ratio in the tested red mud-based stabilized soil contributes to the improvement of the strength of the red mud-based stabilized soil.

## 5. Conclusions

In this experiment, the stabilized soil material was prepared by modifying the red mud of aluminum industrial waste; the strength and microscopic characteristics of the red mud-based stabilized soil with different modified materials and different amounts of modified materials were tested, and the curing mechanism was analyzed, and the main findings were as follows:

(1) Cement alone can improve the unconfined compressive strength of red mud-based stabilized soil; with the synergistic modification of nano-SiO_2_, gypsum, and cement, the 7 d unconfined compressive strength of red mud-based stabilized soil is greater than 2 MPa under the synergistic effect of nano-SiO_2_ (1%, 2% and 3%, respectively), which meets the compressive strength requirement of road subgrade material, and the highest unconfined compressive strength of nano-SiO_2_ combination is 2748 kPa;
(2) In the microstructure study, the SEM test results showed that the soil structural compactness did not increase with the increase in nano-SiO_2_ when nano-SiO_2_, gypsum, and cement were co-modified, and the soil structural crack rate was the lowest, and the structural compactness was the best when nano-SiO_2_ was used at 1%;
(3) High magnification SEM tests reveal that when nano-SiO_2_, gypsum, and cement are synergistically modified, it is found that the increase in needle-like and columnar AFt in the cementitious products is due to the 6% gypsum added to the modified material, which creates conditions for the formation of more Aft. The XRD results showed that the gypsum diffraction peaks of the NS1CS6PC3 modified combination of red clay-based stabilized soil tended to disappear with the growth of the maintenance age, indicating that it was continuously transformed into AFt. The increase in binding energy of hydration product-related ions in the modified material also indicates that the strength of the modified material is improved;
(4) Mechanical compaction is a prerequisite for chemical curing, and the chemical curing mechanism contains the hydration reaction, pozzolanic reaction, the promotion effect of nano-SiO_2_, and the enhancement effect of gypsum. The amount of nano-SiO_2_ is small, but it can promote the early hydration reaction process and hydration degree, providing more silica sources for the stabilized soil material. This paper established a model of nano-SiO_2_ to promote the early hydration process of red clay-based stabilized soil and revealed the mechanism of nano-SiO_2_ to promote the hydration process of red clay-based stabilized soil; the modification effect of gypsum is key in providing a calcium source to the red clay-based stabilized soil system. The key role of gypsum in the modification is to provide a calcium source, which contributes to the conversion of all or most of the C3A in cement into AFt, and at the same time, with Si^4+^ ions in the material, to generate C-S-H hydration gel under alkaline environment, which further enhances the strength of the modified stabilized soil.

## Figures and Tables

**Table 1:** Main chemical components of red mud.

	Fe_2_O_3_	Al_2_O_3_	SiO_2_	CaO	Na_2_O	TiO_2_	MgO	K_2_O	LOL
Average value	37.08%	18.89%	12.35%	10.56%	5.23%	6.21%	0.68%	0.12%	6.23%

**Table 2:** Nano-SiO_2_ technical parameter table.

Materials	Particle Size (nm)	Purity (%)	Specific Surface Area (m^2^/g)	Bulk Density (g/cm^3^)	Color	pH
Nano-SiO_2_	1~100	99.9	240	0.06	White	4–7

**Table 3:** Main chemical components of cement.

Calcium Oxide (CaO)	Silicon Dioxide (SiO)_2_	Aluminum Oxide (AlO)_23_	Iron Oxide (FeO)_23_	Titanium Dioxide (TiO)_2_	Sulfur Trioxide (SO)_3_
65.13%	21.32%	5.35%	3.96%	0.25%	0.30%

**Table 4:** Collaborative combination scheme table.

Modified Solutions	I	II	III	IV	V
PC individually modified	PC1	PC3	PC5	PC7	PC9
NS+ CS6 + PC3 synergistic modification	NS0.5CS6PC3	NS1CS6PC3	NS2CS6PC3	NS3CS6PC3	

**Table 5:** Binding energy table of main elements of different modified samples at different curing ages.

Modified Specimens	Conservation Age/d	Binding Energy/eV					
		Ca2p	Si2p	Al2p	Na1s	O1s	S2p
NS1CS6PC3	7	346.90	102.17	74.14	1071.71	531.32	169.09
	28	346.97	102.18	74.18	1071.76	531.33	169.28
	60	347.05	102.23	74.23	1071.94	531.44	169.61
NS2CS6PC3	60	347.04	102.47	74.22	1071.72	531.47	169.55
NS3CS6PC3	60	346.96	102.65	74.32	1071.63	531.58	169.50

## Data Availability

Some or all data, models, and code that support the findings of this study are available from the corresponding author upon reasonable request.
